# Sequence and Structural Characterization of Toll-Like Receptor 6 from Human and Related Species

**DOI:** 10.1155/2021/5545183

**Published:** 2021-04-10

**Authors:** Ghulam Mustafa, Hafiza Salaha Mahrosh, Rawaba Arif

**Affiliations:** Department of Biochemistry, Government College University, Faisalabad 38000, Pakistan

## Abstract

Toll-like receptors (TLRs) play an important role in the innate immune response against various pathogens. They serve as expected targets of natural selection in those species which are adapted to habitats with contrasting pathogen burdens. Till date, sufficient literature about TLRs especially TLR6 is not available. The current study was therefore planned to show evolutionary patterns of human TLRs generally and TLR6 specifically along with their conservation and diversity. The study also deals with characteristic polymorphic patterns of TLR6 in humans which are involved in serious clinical consequences. The sequence analysis of TLR6 from different mammals revealed conserved regions in the protein sequence. With respect to TLR6 evolution, human showed a close evolutionary relationship with chimpanzee and orangutans, while monkeys were appeared in a separate clade showing a distant evolutionary relationship. Old World monkeys and New World monkeys made their separate clades but both have evolved from a common ancestor. The C-terminal of human TLRs (TLR1 to TLR10) exhibited more conservation as compared to other regions. The phylogram of human TLRs showed that TLR6 is closely related to TLR1 and both TLRs shared a common ancestor with TLR10. The domain analysis has revealed that TLR1 and TLR10 have least (i.e., 4) number of leucine-rich repeat (LRR) while TLR6 contains five LRRs. Three single nucleotide polymorphisms were found in TLR6 which were found to be associated with benign. Conclusively, the current comparative sequence analyses and phylogenetic analyses provided informative insights into the process of TLR evolution in mammals. Furthermore, the polymorphism analysis would serve as a useful marker in the early detection of susceptibility and resistance against cancers and other diseases in humans.

## 1. Introduction

The host immunity begins with first line of defence including skin, endothelium, lysozyme, saliva, gastric juice, and mucous membranes that prevent foreign infectious agents to reach into the cellular potential sites to cause infections. The second and third lines of defense are comprised of nonspecific resistance (innate immunity) and specific resistance (acquired immunity), respectively. The former relies on inflammations and activation of phagocytic cells, and the latter is cell-mediated immunity linked with antibody production and activation of antigen-sensitized cytotoxic T cells. The professional T cells, B cells, leukocytes, phagocytes, and release of cytokines play a central role in the cellular immunity by neutralizing the threats [1].

Cell-mediated immunity plays a critical role in combating multiple types of infections and pathogenic attacks. Cell-mediated innate immunity is nonspecific to invaders, dependent on the phagocytes and proteins to recognize the conserved regions of pathogen-associated molecular patterns (PAMPs) and/or pathogenic endogenous damage-associated molecular patterns (DAMPs) to destroy them quickly [2]. The innate immunity is mainly involved the recognition of DAMPs and PAMPs via pattern recognition receptors (PRRs) [3]. Toll-like receptors (TLRs) are type I glycoproteins that belong to the family of PRRs and respond towards the special repertoire of pathogenic molecules including PAMPs and DAMPs. RLRs are pattern recognition receptors with 10-27 extracellular leucine-rich repeats, intracellular cytoplasmic Toll/interleukin-1 receptor (TIR) domain, and a single transmembrane helix, and these are the structurally conserved regions of TLRs [4]. To date, 10 human TLRs (TLRs 1-10) and 13 murine TLRs (TLRs 1-9 and TLRs 11-13) have been reported. The TLR10 has been found to be nonfunctional in mouse [5].

Toll-like receptors are expressed on all innate immunity cells including macrophages, basophils, neutrophils, natural killer cells, and dendritic cells as well as on adaptive immune cell lymphocytes (T and B cells) [6]. The cellular repair mechanism is activated by activation of TLRs that stimulate the signalling cascade of host immune system which helps the release of immune modulators and cytokines [7]. The signalling cascade of TLRs depends on the nature of stimuli and is regulated by two distinct pathways (i.e., TIR-domain-containing adapter-inducing interferon-*β*- (TRIF-) dependent pathway and MyD88-dependent pathway). TLR3 and TLR4 utilize the TRIF-dependent pathway that leads towards the stimulation of type 1 interferons, and all TLRs except TLR3 utilize MyD88-dependent pathway associated with inflammatory cytokine production [6]. The overexpression of TLRs drastically alters the homeostasis of immune system by sustaining the levels of proinflammatory cytokines, and type 1 interferon contributes in the development and progressions of autoimmune diseases including lupus erythematous, rheumatoid arthritis, Alzheimer's disease, and type I diabetes mellitus [6].

TLR6 forms a heterodimer with TLR2 to broaden the ligand capacity against different pathogens. The mutations in TLR1, TLR2, and TLR6 lead towards the progression of different autoimmune disorders. Different studies have supported the evidence of involvement of TLR6 in the progression of many autoimmune complications including sepsis, coronary artery disease, and intestinal inflammation [8–10]. Therefore, in current study, the evolutionary and genetic level study of TLR6 was conducted which will provide substantial knowledge about the variants and polymorphisms associated with different disorders. In this study, we have adopted computational biology mode of study to explore polymorphic residues and estimated evolutionary relationships of TLR6 among various mammals and conservation of human TLRs.

## 2. Materials and Methods

### 2.1. Retrieval of Human TLR6 Protein Sequence

The human TLR6 protein sequence was retrieved from NCBI's Entrez protein database with accession number: AAY88762.1 and analysed using PSI-BLAST (Position-Specific Iterative Basic Local Alignment Search Tool) [11] available on the NCBI website (http://www.ncbi.nlm.nih.gov/).

### 2.2. Phylogenetic Analysis

Along with human TLR6 protein sequence, forty most similar reported sequences of TLR6 from different mammals were also retrieved from protein database for phylogenetic systematics. The expected threshold value was set to 0.05 in PSI-BLAST, and only those sequences were selected which showed *E*-value better than threshold. All sequences were aligned using ClustalX and imported into the MEGA7 program [12] for manual alignment. Neighbor-joining (NJ) phylogenetic tree was reconstructed using MEGA7 with 100 bootstrap replicates [13].

### 2.3. Comparative Sequence Analysis and Domain Organization of Human Toll-Like Receptors

The protein sequences of all TLRs (i.e., TLRs 1-10) from humans were retrieved from NCBI's Entrez protein database to reveal their diversity and conservation at amino acid level. Multiple sequence alignment of protein sequences of all TLRs was performed through Geneious [14] for comparative study. An NJ phylogenetic tree of all human TLRs was also reconstructed using MEGA7 with 100 bootstrap replicates to explore their evolutionary relationships. To identify conserved protein domains in human TLRs, the SMART server (http://smart.embl-heidelberg.de/) was used with the default parameters [15].

### 2.4. Allelic Distribution and Polymorphism of Human TLR6 Gene

The database of single nucleotide polymorphism (dbSNP) of NCBI was explored to reveal allelic distribution and TLR6 polymorphism associated with diseases. The dbSNP contains single nucleotide variations, microsatellites, and small-scale insertions and deletions in humans. Only those SNPs of human TLR6 were selected in this study which exhibited clear clinical significance.

## 3. Results

**Figure 1:**
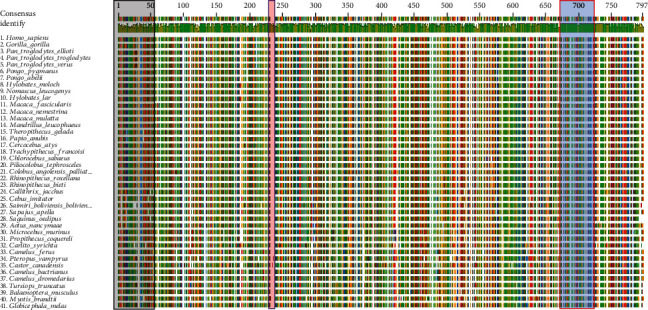
Multiple sequence alignment of TLR6 from selected organisms to show sequence similarities/differences. Similar color bars are showing similar sequences. Red highlighted areas are showing regions present in the large flying fox (*Pteropus vampyrus*) and the North American beaver (*Castor canadensis*) but not in other mammals. Highly variable regions are depicted as black rectangle. The blue highlighted region is highly agreed with the consensus.

BLAST has been the most popular algorithm for similarity search that can accommodate nucleotide or protein sequences. BLAST was used to identify local regions of similarity and statistical significance of TLR6 protein sequences from selected organisms. Geneious was employed to perform multiple sequence alignment (Figure 1). The truncated sequences were deleted, and longer sequences were shortened in the multiple sequence alignment to make them all equal in length. The consensus sequence is also shown above the alignment. To show identity among TLR6 sequences, bars are shown in the alignment. Higher the green bar in the alignment, higher is the identity among those regions. The comparative sequence analysis is showing greater diversity among TLR6 protein sequences among selected organisms in the regions of amino acids 1 to 100. High sequence conservation is revealed in the regions of 640 to 790.

### 3.1. Analysis of TLR6 Phylogenetic Tree

**Figure 2:**
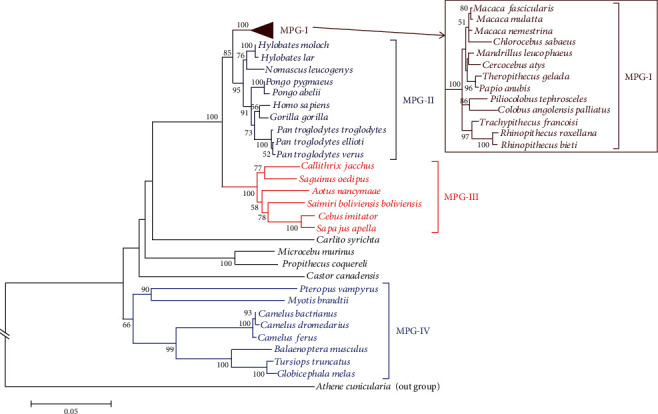
Phylogenetic relationships of TLR6 from human and other homologs. The phylogram is labelled on the basis of their monophyletic groups. The protein sequence of TLR1 from *Athene cunicularia* was used as an out-group.

To establish the evolutionary relationships, a phylogenetic tree of TLR6 protein from human and forty members of mammals with maximum identity was reconstructed (Figure 2). The evolutionary history was inferred using the neighbor-joining method [16]. The percentages of replicate trees in which the associated taxa clustered together in the bootstrap test (100 replicates) are shown next to the branches [17]. The tree is drawn to scale, with branch lengths in the same units as those of the evolutionary distances used to infer the phylogenetic tree. The evolutionary distances were computed using the Poisson correction method [18] and are in the units of the number of amino acid substitutions per site. Overall mean distance was found to be 0.143 that shows estimates of average evolutionary divergence over all sequence pairs.

The phylogram was fully resolved into different clades. The phylogram has been divided into four different monophyletic groups (i.e., MPG-I, MPG-II, MPG-III, and MPG-IV) based on cladding patterns of all taxa. *Homo sapiens* was appeared in MPG-II and showed close evolutionary relationships with gorilla and chimpanzee. Two orangutan species (i.e., *Pongo pygmaeus* and *Pongo abelii*) also appeared in this clade. The latter is one of the two species of Asian ape, while other orangutan species belong to rainforests of Borneo and Sumatra. Three gibbon species also joined the MPG-I (*Hylobates moloch*, *Hylobates lar*, and *Nomascus leucogenys*) showing their evolutionary closeness with other taxa in the group.

The MPG-I was found to be a clade of monkeys. Long-tailed or crab-eating macaque (*Macaca fascicularis*), rhesus macaque (*Macaca mulatta*), and southern pig-tailed macaque (*Macaca nemestrina*) were found to be closely related species, while all three distantly related to the sabaeus monkey (*Chlorocebus sabaeus*). The drill (*Mandrillus leucophaeus*) showed a close evolutionary relationship with the sooty mangabey (*Cercocebus atys*); whereas, gelada (*Theropithecus gelada*) and the olive baboon (*Papio anubis*) were found to be related species. Similarly, the Ashy red colobus monkey (*Piliocolobus tephrosceles*) and angolan colobus monkeys (*Colobus angolensis palliates*) showed close evolutionary relationship. The leaf monkey or langur (*Trachypithecus francoisi*) was distantly related to two related species which were golden monkey (*Rhinopithecus roxellana*) and the black snub-nosed monkey (*Rhinopithecus bieti*).

The MPG-III was consisted of New World monkeys which are small to mid-sized primates. A node with 100 bootstrap value is showing that the New World monkeys and Old World monkeys shared a common ancestor. The Philippine tarsier (*Carlito syrichta*) and the North American beaver (*Castor canadensis*) both did not appear in any clade and showing divergence from other monophyletic groups. Two lemur species, the gray mouse lemur (*Microcebus murinus*) and coquerel's sifaka (*Propithecus coquereli*), exhibited a strong evolutionary relationship.

The fourth clade (i.e., MPG-IV) was consisted of diverse group of taxa. The large flying fox (*Pteropus vampyrus*) and a Brandt's bat (*Myotis brandtii*) were found to be evolutionary related species. Three *Camelus* species also appeared in this clade. Then, some marine organisms such as blue whale (*Balaenoptera musculus*), the common bottlenose dolphin or Atlantic bottlenose dolphin (*Tursiops truncatus*), and the long-finned pilot whale (*Globicephala melas*) also joined this clade with strong evolutionary relationships to each other. The protein sequence of TLR1 from a burrowing owl (*Athene cunicularia*) was used to root the tree.

### 3.2. Conservation and Diversification of Human TLRs

**Figure 3:**
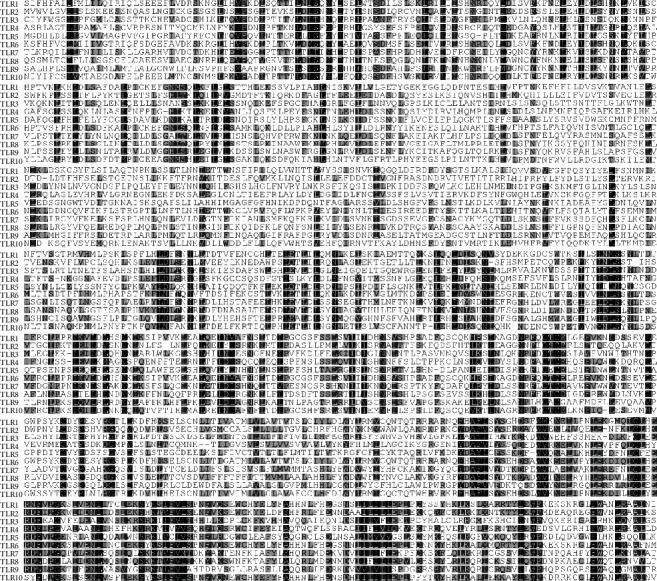
Multiple sequence alignment of human TLRs (TLR1 to TLR10). The dark shaded areas are showing conservation among various TLRs (more the intensity of the dark shaded area, higher is the conservation in that region). The conservation scale: black = conserved, gray = average, and white = variable.

The protein sequence of human TLR6 was compared with other human TLRs (TLRs 1, 2, 3, 4, 5, 7, 8, 9, and 10), and their multiple sequence alignment is shown in Figure 3. More sequence conservation has been depicted at C-terminal of the TLR proteins. Dark shaded areas are highly conserved, light shaded areas are somewhat conserved, while unshaded areas are showing diversification among TLR sequences.

The sequence logo of human TLRs is shown in Figure S1. In logo, the conserved sequences are represented by large single-letter codes of amino acids at specific positions. The sequences which are missing from other TLRs are highlighted by pink rectangles.

### 3.3. Evolutionary Relationships and Domain Organization Analysis of Human TLRs

**Figure 4:**
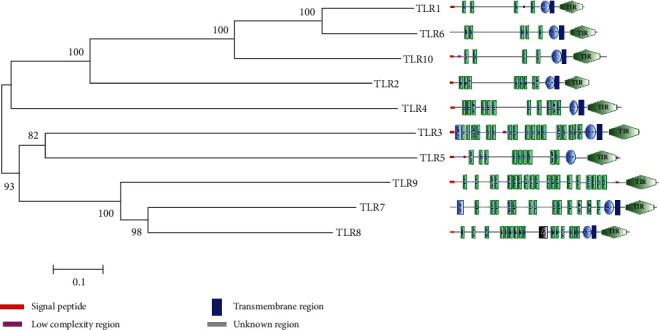
Phylogenetic tree of human TLRs. The evolutionary history was inferred using the NJ method. The bootstrap consensus tree inferred from 100 replicates is taken to represent the evolutionary history of the taxa analysed. The evolutionary distances were computed using the Poisson correction method and are in the units of the number of amino acid substitutions per site. Different colors and shapes are representing different domains and regions.

The multiple sequence alignment of human TLRs was further used to reconstruct a phylogenetic tree (Figure 4). Interesting relationships among human TLRs were revealed from this phylogram. The TLR6 was found to be closely related to TLR1 as both have evolved from a common ancestor that is supported by 100 bootstrap value at their ancestral node. Both TLRs further shared a common ancestor with TLR10 and showing their evolutionary relationship with TLR10. TLR1 and TLR10 showed minimum number of leucine-rich repeat (LRR) domains and also both have signal peptides, while TLR6 contains five LRR domains and also does not have a signal peptide. TLR2 and TLR4 are showing distant evolutionary relationships with TLR1, TLR6, and TLR10. A high bootstrap value (82%) on the ancestral node of TLR3 and TLR5 is showing that both TLRs have evolved in a close evolutionary relationship. TLR7 and TLR8 shared a common ancestor and showing that both are closely related which is supported by a high bootstrap value of 98%, and both TLRs further joined the ancestral node of TLR9 showing an evolutionary relationship with TLR9. It has been depicted by a high bootstrap value (93%) that TLR3 and TLR5 have close evolutionary relationship and shared a common ancestor with TLR9, TLR7, and TLR8 which is showing that a distant evolutionary relationship is present between both groups of human TLRs. TIR domain was found in all TLRs, while TLR3 and TLR9 showed maximum number of domains (i.e., 20). LRR carboxyl-terminal (LRR_CT) domain was not found in TLR7. LRR amino-terminal (LRR_NT) domain was found only in TLR3 and TLR7.

### 3.4. Comparative Sequence Analysis of Human TLR6 and TLR1

**Figure 5:**
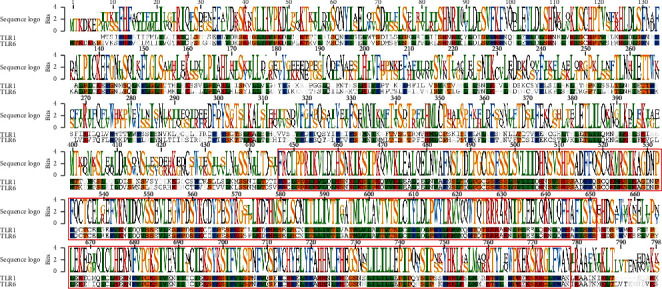
Comparison of human TLR6 and TLR1 protein sequences. Red rectangles are showing conservation between both sequences. Sequence logo has been generated on the basis of sequence conservation and shown above the alignment.

Based on phylogenetic analysis, the human TLR6 showed a strong evolutionary relationship with TLR1. Therefore, a sequence logo was generated through comparative sequence analysis of both proteins to access similarity between both proteins (Figure 5). The sequence logo is shown above the aligned sequences. The amino acids in their single-letter codes are shown in the sequence logo. Higher the size of the single-letter code of amino acids, higher the conservation is at that position. A high similarity between both sequences has been labelled in red rectangles. It has been revealed that both sequences are not highly similar in the region of 1 to 145.

### 3.5. SNPs of Human TLR6 Gene with Clinical Significance

The single nucleotide polymorphisms (SNPs) of human TLR6 gene with serious clinical significance are given in Table 1. Only the SNPs with known clinical significances were added. A total of four SNPs have been found in dbSNP that are associated with benign. Three variants of single nucleotide variations and one variant of deletion were found in these SNPs. In first SNP, the thiamine (T) is replaced with cytosine (C) and the highest frequency of the alternate allele (C) has been found in African American. In second SNP, the cytosine (C) was the reference allele in TLR6 gene which is altered with thiamine (T) which causes a missense mutation. The highest frequency of the alternate allele (T) has been found in Europeans. Third SNP was also a SNV, and in this polymorphism, cytosine (C) has been altered with adenine (A) and causes a missense mutation with its highest frequency in South Asia. The last SNP was found to be a deletion in which adenine and thiamine (AT) were deleted and caused in frameshift mutation.

## 4. Discussion

The inflammatory responses have been closely associated with TLR6-mediated extracellular signal-regulated kinase (ERK) and p38 pathways in macrophages [19]. The onset of single nucleotide polymorphisms (SNPs) in TLR6 encoding gene or protein leads towards alteration in the functioning of this PRR. The polymorphic residues on the amino acid sequence of TLRs result in SNPs that alter the inflammatory signalling cascade associated with different disorders [20]. In this study, we have demonstrated the phylogenetic relationships of TLRs specifically TLR6 reported in humans. In this phylogram, the TLR6 of human showed a strong evolutionary relatedness with gorilla. Woodman et al. [21] also conducted a phylogenetic analysis of TLR6 from different mammals including primates. In their phylogram, the human also showed a strong evolutionary relationship with chimpanzee and gorilla. In another study, Soni et al. [22] reconstructed a phylogenetic tree of DNA sequences of different TLRs (TLR1 to TLR10) of various vertebrates. They found that TLR2, TLR6, TLR9, and TLR10 were revealed to be conserved during the entire course of their evolution.

Phylogeny is an effective tool for determining the evolutionary relationships among various species in terms of protein and its functionality [23, 24]. To further elucidate the evolutionary relationships of TLRs, a phylogram among all human TLR proteins was also reconstructed. It was revealed that TLR6 is closely related to TLR1, and both TLRs were emerged from the ancestral node of TLR10. Similar findings were reported by Kumari and Singh [25] who conducted phylogenetic analysis of TLR6 gene fragment of different animals. The TLR6 was found nearest to TLR1 and TLR10 mRNA of the Sus scrofa. The results of Banerjee et al. [26] are also in accordance to these findings. They have reported that genes of TLR1, TLR6, and TLR10 might have arisen from a gene duplication event which could be evidently proved by the presence of all three genes on the same chromosome (BTA6) and also all three genes belong to the same family of TLR2. TLR2 and TLR4 appeared in the same clade in this study. Tantia et al. [27] also reported that both genes are highly conserved across all mammalian species.

In mammals, the pattern recognition receptors have distinct classes including RIG-I-like receptors (RLRs), Toll-like receptors (TLRs), NOD-like receptors (NLRs), AIM2-like receptors (ALRs), and C-type lectin receptors (CLRs) which trigger the immediate host defence that leads to the induction of innate immunity responses [5, 28]. Among all the discovered PRRs, TLRs extensively initiate the survey of recognition of self- and non-self-antigens [29]. Toll-like receptors are generally expressed on cellular surface or in endosome in the dimer form, preferentially as homodimers or heterodimers. Among the reported Toll-like receptors, TLRs 1-2 and TLRs 4-6 are expressed on cell membrane, while TLR3, TLR7, TLR8, and TLR9 are localized in endosomes [30]. The TLRs are mainly associated with invader recognition features via eternal PAMPs of pathogens. These ligands can be characterized into three types, i.e., proteins are recognized by TLR5, TLR3, TLR7, TLR8, and TLR9; nucleic acids are recognized by TLR5; and lipids are recognized by TLR2/TLR6, TLR2/TLR1, and TLR4, respectively [28].

Each innate immune cell contains special TLRs that mediate in cellular immunity by recognizing the specific PAMP/DAMP and inducing proinflammatory cytokines. The intracellular Toll/interleukin-1 (IL-1) receptor (TIR) domains of TLRs play a leading role in the regulation of downstream signalling cascade [4]. TLRs reinvigorate adapter proteins that act as a platform for activations of IL-1R-associated protein kinases (IRAK) 1, 2, and 4 and TNF receptor-associated factor 6 (TRAF6) which translocate the NF-*κ*B, proinflammatory transcription factor, IRF3, and AP-1. Each transcriptional factor transcribes specific genes to encode different proteins such as proinflammatory cytokines, chemokines, antimicrobial peptides, and type 1 interferon.

The differences in producing immune molecules or polymorphisms in TLRs not only exhibit substantial influence on responses to a wide range of pathogens but are also associated with susceptibility and resistance to different diseases. In this study, a total of four SNPs were found in human TLR6 gene with serious clinical significance and result in the development of benign. In a study, Elmaghraby et al. [31] have reported two novel nonsynonymous SNPs in TLR6 which were resulted from transversions. In another study, Mariotti et al. [32] found 855G>A SNPs and 2315T>C SNPs in the TLR6. In a study of prostate cancer, nine TLR6 SNPs were reported by Sun et al. [33]. Similarly, Noreen and Arshad [34] have reported that a C>T SNP (C745T) is associated with asthma and colitis, and an A>G SNP (A1401G) is associated with an increased risk of prostate cancer. Another SNP A>C, G,T (rs5743810) affects the expression and function of TLR6. The change in amino acid from Ser to Pro reduces the function of TLR6 and weakens the regulation of innate immune system in predisposed individuals. This mutation can potentially develop cancer [35]. The polymorphisms in the coding regions are believed to be involved in cancer development because of their roles in gene expression regulation. It has been found that TLR6 functionally interacts with TLR2 for mediating the cellular response against bacterial lipoproteins and causes the activation of NF-*κ*B pathway and inflammatory events. This activation further contributes towards tumor development and progression [36]. In spite of its enhanced expression and activation, the function and role of TLR6 in cancer are still not clearly understood. Identification of single nucleotide polymorphism (SNP) in genes involved with the innate immune response can be a useful marker in early detection of resistance or susceptibility in humans.

## 5. Conclusion

The genes encoding TLRs play an important role in the early defense against pathogens. The evolutionary history of TLR6 and evolutionary relationships of human TLRs were studied through phylogenetic analyses. Direct sequence comparisons and standard evolutionary approaches were employed to determine sequence conservation and diversity in human TLRs and TLR6 between human and other closely related species. In addition, the comparison of patterning of human TLR domains was performed. Single nucleotide polymorphisms involved in the development of benign were also revealed from human TLR6. These findings will facilitate the further exploration of TLRs especially TLR6 roles in regulating the immune system of human.

## Figures and Tables

**Table 1:** Distribution, polymorphism, and clinical significance of alleles of human TLR6 gene.

Sr. no.	Reference SNP	Variant type	Alleles	Functional consequence	Clinical significance	Allele frequency (global)^†^		Population with highest Alt allele frequency
						Ref allele	Alt allele	
1	rs5743812	SNV	T>C	Coding sequence variant, synonymous variant	Benign	*T* = 0.99731	*C* = 0.00269	African American (*C* = 0.0364)
2	rs5743816	SNV	C>T	Missense variant, coding sequence variant	Benign	*C* = 0.87967	*T* = 0.12033	European (*T* = 0.14193)
3	rs75244616	SNV	C>A	Missense variant, coding sequence variant	Benign	*C* = 0.99933	*A* = 0.00067	South Asian (*A* = 0.05)
4	rs863223364	DEL	AT>-	Frameshift variant, coding sequence variant	Benign	NA	NA	NA

SNV: single nucleotide variation; DEL: deletion; NA: not available. ^†^The ALFA project provides aggregate allele frequency from database of Genotypes and Phenotypes (dbGaP).

## Data Availability

The data used to support the findings of this study are available from the corresponding author upon request.

## Supplementary Materials

Supplementary Materials Figure S1: sequence logo of human TLRs. In logo, the conserved sequences are represented by large single-letter codes of amino acids at specific positions. The sequences which are missing from other TLRs are highlighted by pink rectangles.
