# Supplementary material for: Sequence and Structural Characterization of Toll-Like Receptor 6 from Human and Related Species
Source: Biomed Res Int. 2021 Apr 10;2021:5545183. doi: 10.1155/2021/5545183 (PMC8055411; doi:10.1155/2021/5545183)
Supplement: Supplementary Materials — Figure S1: sequence logo of human TLRs. In logo, the conserved sequences are represented by large single-letter codes of amino acids at specific positions. The sequences which are missing from other TLRs are highlighted by pink rectangles. [file 5545183.f1.docx]

**Sequence and structural characterization of toll-like receptor 6 from human and related species**


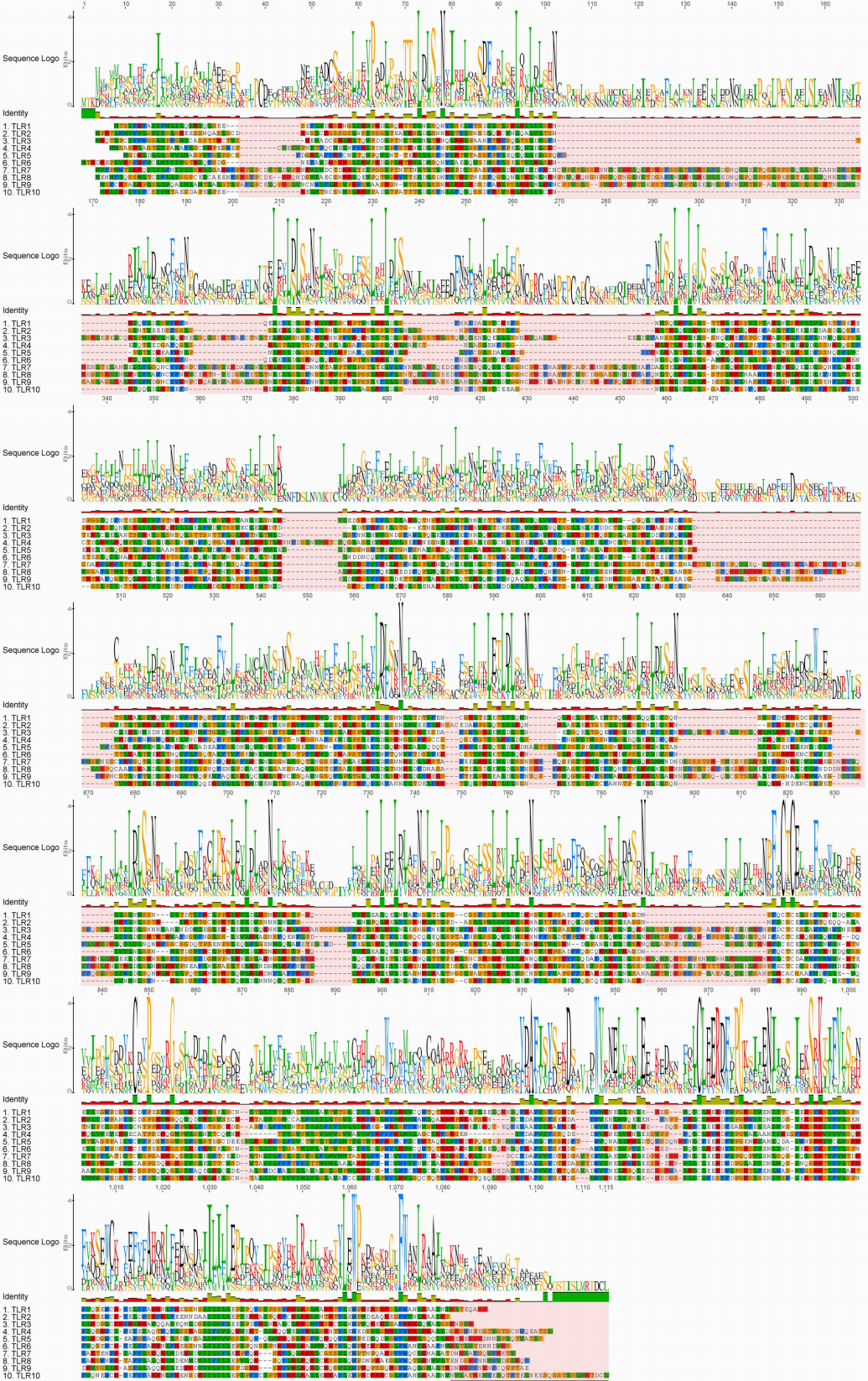


**Fig S1: Sequence logo of human TLRs**. In logo, the conserved sequences are represented by large single letter codes of amino acids at specific positions. The sequences which are missing from other TLRs are highlighted by pink rectangles
